# Detection of virus mRNA within infected host cells using an isothermal nucleic acid amplification assay: marine cyanophage gene expression within *Synechococcus *sp

**DOI:** 10.1186/1743-422X-4-52

**Published:** 2007-06-06

**Authors:** Susan D Wharam, Matthew J Hall, William H Wilson

**Affiliations:** 1 Bigelow Laboratory for Ocean Sciences, 180 McKown Point, West Boothbay Harbor, Maine 04575, USA; 2 Cytocell Ltd., Banbury Business Park, Adderbury, OX17 3SN, UK; 3 Marine Biological Association, Citadel Hill, Plymouth, PL1 2PB, UK; 4 Plymouth Marine Laboratory, Prospect Place, The Hoe, Plymouth, PL1 3DH, UK

## Abstract

**Background:**

Signal-Mediated Amplification of RNA Technology (SMART) is an isothermal nucleic acid amplification technology, developed for the detection of specific target sequences, either RNA (for expression) or DNA. Cyanophages are viruses that infect cyanobacteria. Marine cyanophages are ubiquitous in the surface layers of the ocean where they infect members of the globally important genus *Synechococcus*.

**Results:**

Here we report that the SMART assay allowed us to differentiate between infected and non-infected host cultures. Expression of the cyanophage strain S-PM2 portal vertex gene (g20) was detected from infected host *Synechococcus *sp. WH7803 cells. Using the SMART assay, we demonstrated that g20 mRNA peaked 240 – 360 minutes post-infection, allowing us to characterise this as a mid to late transcript. g20 DNA was also detected, peaking 10 hours post-infection, coinciding with the onset of host lysis.

**Conclusion:**

The SMART assay is based on isothermal nucleic acid amplification, allowing the detection of specific sequences of DNA or RNA. It was shown to be suitable for differentiating between virus-infected and non-infected host cultures and for the detection of virus gene expression: the first reported use of this technology for such applications.

## Background

The Signal-Mediated Amplification of RNA Technology (SMART, developed by Cytocell Ltd., Banbury, UK), also referred to as CytAMP^® ^(British BioCell International, Cardiff, UK) was originally developed for the medical diagnostics industry [1]. Public Health Laboratory trials have compared CytAMP^® ^with more conventional methods for the specific detection of MRSA (methicillin-resistant *Staphylococcus aureus*) [2]. A review, outlining guidelines for the laboratory diagnosis and susceptibility testing of MRSA, reported that the sensitivity and specificity of CytAMP^® ^was comparable to those of PCR for this purpose [3].

**Figure 1:**
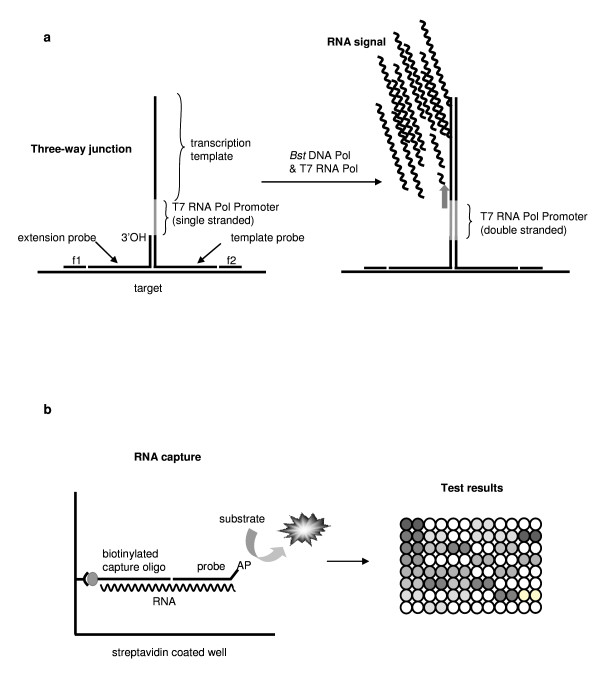
The SMART assay. (**a**) Specific probes hybridise with the target to form a three-way junction (3WJ), assisted by facilitator probes (f1 & f2). The 3WJ initially contains a single-stranded, inactive T7 RNA polymerase promoter sequence. The promoter is made double stranded (active) by extension (by *Bst *DNA polymerase) off the 3' of the extension probe, leading to the generation of large amounts of RNA signal (by T7 RNA polymerase), which may itself be amplified if required. (**b**) Detection of RNA signal by ELOSA (Enzyme Linked OligoSorbant Assay). The assay uses 2 specific probes: a biotinylated capture probe and enzyme (Alkaline phosphatase, AP) linked detection probe. Non-specific nucleic acid and 3WJ probes are removed, following binding in a streptavidin coated well, and RNA signal is detected via a colour change. Quantification of signal takes place in a 96 well plate, allowing multiple samples to be analysed simultaneously.

The SMART assay, summarised in figure 1, has been described in detail elsewhere [1,4]. Briefly, the assay uses two oligonucleotide probes which hybridise specifically to the target, at adjacent sites, and also to each other to form a "T" structure known as a three-way junction (3WJ) (Fig. 1a). The efficiency of 3WJ formation is greatly enhanced by the use of facilitator probes that anneal to the target adjacent to the 3WJ. Only when specific target nucleic acid is present, a T7 RNA polymerase promoter sequence within the 3WJ structure becomes double stranded, and hence activated. T7 RNA polymerase then produces large amounts of an RNA transcript. This RNA is the assay signal and it can be further amplified by the same process if required, and detected by an enzyme-linked oligosorbant assay (ELOSA) (Fig. 1b). Amplification and signal detection processes have been fully described and explained previously [1,4].

Here, we report the first application of this isothermal nucleic acid amplification assay for the detection of viral DNA and RNA within infected host cells. This is also the first report of the assay being used to detect gene expression.

The viruses chosen for this study were cyanophages. These are viruses that infect cyanobacteria, which are globally important photosynthetic microorganisms. Cyanophages have a wide spectrum of host ranges, are ubiquitous and can be easily isolated from a range of aquatic environments [5]. Marine cyanophages are extremely numerous in surface seawater [6-9]. Their hosts, *Synechococcus *spp., are marine cyanobacteria, which also have a widespread distribution throughout the world's oceans and are thought to contribute up to 25% of primary productivity in the open ocean [10]. There is great interest in marine cyanophages, as they are key components of microbial communities and influence host populations [11] and biogeochemical cycling [12-14], as well as primary productivity.

Much of the emphasis of research on marine cyanophages has focussed on the dynamics (or propagation strategy) between phage and host *in situ *and on determining their genetic diversities [15-20]. Until recently, very little had been reported about marine cyanophage gene expression, gene function or phage assembly apart from what could be deduced from sequence information [21-25]. However, following the discovery of photosynthetic genes in marine cyanophages [26-28], studies on their expression using microarrays [29] and quantitative real-time PCR [30], were used to help determine functionality (see review by Clokie et al [31]).

Cyanophage strain S-PM2 was originally isolated by plaque assay from coastal water off Plymouth, UK and belongs to the family Myoviridae, a group of double-stranded DNA phages with contractile tails. S-PM2 has been classified into a sub-group of phages termed the 'exo T-evens' based on a phylogenetic analysis of the structural components, encoded on a 10 kb module, from a range of T-even phages, [22]. One of these structural components is the portal vertex protein (gp20). The g20 gene was originally identified in cyanophages in order to develop a PCR-based assay to analyze natural cyanophage populations [21].

Sequence analysis of g20 in S-PM2 revealed significant similarity to g20 from the enteric coliphage T4, therefore it is likely that the function of gp20 in S-PM2 is similar to that in T4 where it is involved in head assembly. T4 head assembly takes place in several phases and is reviewed extensively in Black *et al*. [32]. Briefly, a prohead is assembled, starting from a membrane-bound initiation complex, the prohead then undergoes proteolysis and is detached from the membrane. The head is then packaged with DNA and final maturation steps occur. At the membrane attachment (proximal) vertex of the prohead shell, there is a dodecameric ring of gp20 protein, termed connector or portal protein. Formation of this structure is essential, and is thought to be the rate-limiting step in T4 prohead initiation. The prohead portal proteins do not undergo proteolysis (as opposed to other prohead proteins which do) and they form the site at which the tail is attached and through which DNA will eventually pass.

The g20 gene is now widely used as a marker to study the diversity and population dynamics of both marine and freshwater cyanophage [19,20,33-38]. Despite such wide scale exploitation of the g20 gene sequence, there have been no previous studies on cyanophage g20 gene expression.

Sequence information from cyanophage g20 was used to develop a set of probes designed for use in the SMART isothermal nucleic acid amplification technology. We have previously reported that the assay discriminated between similar g20 target DNA sequences from two different marine cyanophage strains [4]. Earlier trials also showed the assay, as well as detecting DNA targets, could generate signals from specific RNA (using *E. coli *as a model target organism and a high copy number ribosomal RNA as the target sequence) [1]. The assay conditions are identical, regardless of whether an RNA or DNA target is to be detected.

Here we report that we can detect cyanophage strain S-PM2 g20 mRNA from infected *Synechococcus *sp. WH7803 using a technology based on isothermal nucleic acid amplification. In addition, the SMART assay was used to monitor g20 expression and the subsequent increase in cyanophage DNA in the infected culture. This is the first use of the assay in looking at gene expression, and in detecting viral nucleic acid in an infected host. It is also the first study looking at cyanophage g20 gene expression.

## Results and discussion

### Detection of S-PM2 g20 mRNA from infected host cells

Different sets of SMART probes were designed to detect the coding and non-coding strands of DNA in the S-PM2 g20 target, (Table 1). Probes for the coding strand could generate signal from both DNA and RNA, those for the non-coding strand from DNA only.

**Table 1:** Oligonucleotide probe sequences used in this study.

	Cyanophage target	
	
	S-PM2 g20 coding strand	S-PM2 g20 non-coding strand
Extension probe	TGACCATCGTAAACAAGCTT GTTTCTGTATTCGAAAT	AACAATACTTGCGTGATGTAAT GTCACGTTTTCGAAAT
Template probe	TCGTCTTCCGGTCTCTCCTCT CAAGCCTCAGCGCTCTCTCTC CCTATAGTGAGTCGTATTAATT TCGAAhACGTGACATTACATCA CGCAAGTATTGTTx	TCGTCTTCCGGTCTCTCCTCTCA AGCCTCAGCGCTCTCTCTCCCT ATAGTGAGTCGTATTAATTTCGA AhACAGAAACAAGCTTGTTTACG ATGGTCAAx
Facilitator 1	TGCTTTTTATCATCACGAATC TCTCCTGTTx	ATGTTGGTAATCTACCAAAGGTA AAGGCAGx
Facilitator 2	CTGCCTTTACCTTTGGTAGA TTACCAACAx	ACAGGAGAGATTCGTGATGATAA AAAGCATx

All sequences are written (5' → 3').

S-PM2 GenBank accession number AF016384.

h Indicates position of hexaethylene glycol linker molecule.

x Indicates position of phosphorylation to prevent extension.

Oligonucleotides used for further amplification and detection of the RNA signal are described in Hall *et al*. [4].

A preliminary experiment was performed to determine whether SMART could detect viral RNA from an infected culture. In order to detect S-PM2 g20 mRNA from infected host cells, RNA and DNA were extracted from infected *Synechococcus *sp. WH7803 approximately 24 hours prior to lysis, when viral RNA was predicted to be detectable. Nucleic acid was also extracted from an uninfected culture, for use in controls.

**Figure 2:**
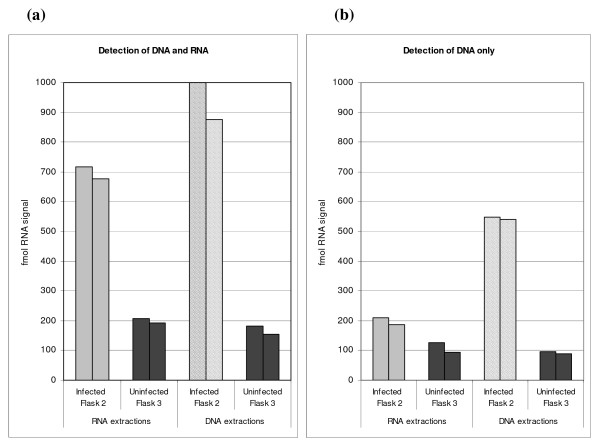
Specific detection of cyanophage S-PM2 g20 target RNA or DNA extracted from infected host *Synechococcus *sp. WH7803. Graphs show signals generated from probes targeting either the coding strand (**a**) (to detect DNA + RNA) or non-coding strand (**b**) (to detect DNA but not RNA). RNA and DNA was extracted from infected cultures grown in flask 2 (24 hours prior to culture lysis). Results are compared to signals generated by both sets of probes using nucleic acid extracted from the uninfected control culture (flask 3). Graphs show the amount of RNA signal (fmol) generated from each target as determined by ELOSA.

Probes designed against the coding strand (to detect DNA + RNA) of g20 generated a SMART assay signal from both DNA and RNA extracted from infected host cells from flask 2 (24 hours prior to culture lysis) (Fig. 2a). Low, background signals were produced from flask 3 (uninfected control). Probes designed against the non-coding strand (to detect DNA but not RNA) of g20 generated a signal from DNA extracted from infected host cells in flask 2 (24 hours prior to culture lysis) (Fig. 2b). Probes for the non-coding strand only produced a very weak signal from the RNA extractions from flask 2 (Fig. 2b). This result confirmed that the coding strand probes were able to detect cyanophage strain S-PM2 g20 mRNA from infected *Synechococcus *sp. WH7803 host cells (Fig. 2a).

### Studying g20 gene expression during the cyanophage infection cycle

**Figure 3:**
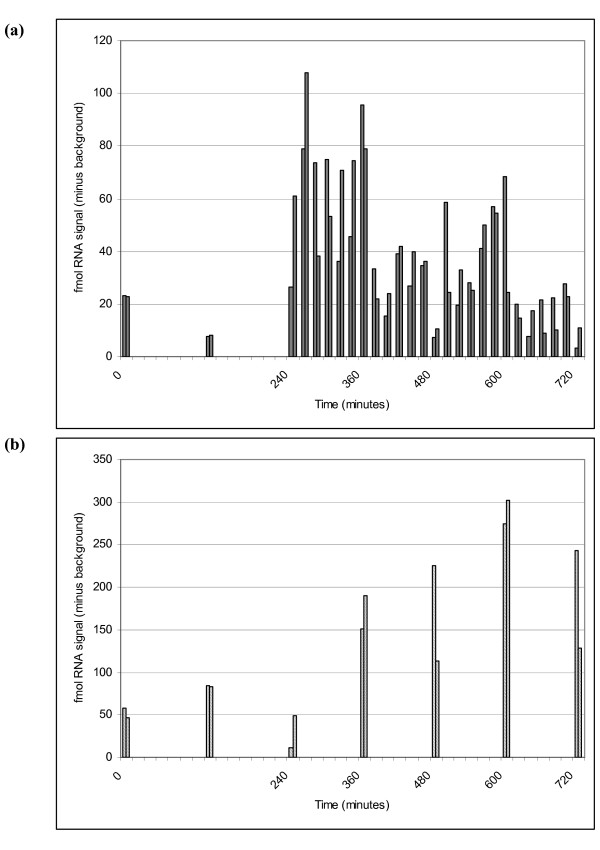
Detection of g20 nucleic acid during infection of *Synechococcus *sp. WH7803 by cyanophage strain S-PM2. Level of cyanophage g20 mRNA (a) and g20 DNA (b) detected from either total RNA (a), or DNA (b), extracted from duplicate samples of infected host cells measured at specific time points (0 – 720 minutes post-infection). Graphs show the amount of RNA signal (fmol) generated from each target as determined by ELOSA.

Further experiments were set up to determine whether the SMART assay could monitor S-PM2 g20 expression during the cyanophage infection cycle. Samples collected over a time series were used to detect changes in the levels of g20 mRNA and DNA following infection of *Synechococcus *by cyanophage S-PM2 (Fig. 3). Results from a preliminary experiment had indicated when the intracellular viral RNA and DNA was likely to peak (i.e. after the 4-hour time point: data not shown), hence the collection of samples increased in intensity from the 4-hour (240 minute) time point. Since the focus was g20 expression, the majority of samples were taken for RNA analysis, but some samples were also analysed for viral DNA, to determine how the sets of data would relate to each other. SMART assays [1,4] were used to detect g20 target mRNA and DNA.

Cyanophage S-PM2 g20 expression was only detected at a low level up to the 240-minute post-infection (i.e. after addition of cyanophages to the host culture) time point. Despite variation in the data, S-PM2 g20 mRNA concentration increased sharply at 240-minutes post-infection, with maximum g20 mRNA detected at 260-minutes post infection (Fig. 3a). At 10 – 11 hours post-infection, g20 mRNA had dropped back to lower concentrations. g20 DNA started to increase 6-hours post-infection, to a maximum level 10-hours post-infection (Fig. 3b).

Data obtained using the SMART assay fit with what is already known about the kinetics of cyanophage infection. In cyanophage strain S-PM2 the onset of lysis occurs after a 9 hour latent period in infected *Synechococcus *sp. WH7803 cells [39]. Maximum g20 expression was observed at 4 hours 20 minutes after infection (Fig. 3a), which is just under half way through the S-PM2 latent period. If compared to phage T4 infection, which has a latent period of 25 minutes [40], this would characterise S-PM2 g20 mRNA as a mid to late transcript. However, recent work by Clokie et al [30] demonstrated that S-PM2 only has 2 (early and late) classes of transcripts rather than the 3 (early, mid and late) observed in T4. In T4, late mRNA is known to direct the synthesis of phage T4 structural proteins as well as proteins that help with phage assembly and are involved in cell lysis. S-PM2, structural genes g18 and g23 were characterised as late transcripts [30] and their expression increased to maximum levels between 4 – 6 hours; this is consistent with S-PM2 g20 (another structural gene) expression data in figure 3 here.

Evidence from electron microscopy and other studies on T4 suggests that the prohead and mature head contain 960 copies of gp23, the major capsid protein, compared with only 12 copies of gp20. Indeed, gp20 is the least abundant of the prohead proteins compared to the others that have copy numbers of between 55 (gp24) and 576 (gp22) [32]. If expression levels are similar in cyanophage S-PM2, it is encouraging that the SMART assay has the necessary sensitivity for detecting g20 gene expression. Therefore, it is likely that the assay would be highly suitable for future expression studies.

The increase in signal from S-PM2 g20 DNA (Fig. 3) is consistent with the continuous replication of cyanophage DNA for eventual packaging into proheads during the infection cycle [41]. The peak of g20 DNA within the host cells 10 hours post-infection is consistent with previous observations that the onset of *Synechococcus *cell lysis occurs from 9 hours post-infection with the burst period continuing to 12 – 15 hours post-infection [39].

## Conclusion

The SMART assay successfully differentiated between infected and non-infected host cultures and detected gene expression. SMART is a simple and sensitive assay, which may be a suitable alternative to more conventional techniques such as Northern analysis and RT-PCR for a range of applications. Also, since is it relatively simple to adapt the assay for the detection of other target sequences, it would be possible to use a set of different specific probes to simultaneously study the expression of different virus and host genes, or assay for different viruses. The equipment used is relatively simple and start up costs low, so for many applications (where there is interest in a relatively small number of genes) it could be developed as a simple alternative to the use of microarrays.

Interest in isothermal nucleic acid amplification is currently increasing. One possible future application of these techniques includes *in situ *work, for example for use in the identification and quantification of infected cells. The repeated rounds of high temperatures involved in thermal cycling can create problems with *in situ *PCR, due to cellular damage. In addition, isothermal amplification techniques are potentially more robust, and have lower energy requirements than methods involving thermal cycling. These are significant benefits for certain applications, such as developing assays for use in remote areas, or for autonomous systems with applications which might include environmental monitoring and assessing public health risks.

The SMART assay, based on isothermal nucleic acid amplification, allows the detection of specific sequences of DNA or RNA. It was shown to be suitable for differentiating between virus-infected and non-infected host cultures and for the detection of gene expression: the first reported use of this technology for such applications.

## Methods

### Cyanophages, host strain and media

Technical details concerning host strain *Synechococcus *sp. WH7803, growth media, culturing, cyanophage strain S-PM2 stock preparation and propagation have been described previously [4,21,39].

### Reagents

Oligonucleotide probes synthesised by phosphoramidite chemistry using a model 380A synthesiser (Applied Biosystems, Foster City, CA, USA) and purified using standard HPLC or FPLC techniques were obtained from Oswel Research products (Southampton, UK).

### Probe design

The sequences of cyanophage-specific probes are listed in table 1. Probes for the S-PM2 g20 coding strand are identical to those used previously [4]. A further set of probes was designed to detect the non-coding strand of g20. The sequences of targets, probes, and RNA signals were designed to minimise potential secondary structure, and their melting temperatures were determined, as described previously [4]. The template probes include a hexaethylene glycol (HEG) linker molecule to reduce non-specific background signal. Sequences of probes for the amplification of signal RNA, capture and detection of SMART signal, and of synthetic product for ELOSA standard curve have all been published previously [1,4].

### Sampling infected host 24 hours prior to lysis

An exponentially growing culture of *Synechococcus *sp. WH7803 was split into 3 × 100 mL aliquots in sterile glass conical flasks and incubated at 25°C under constant illumination (5 to 36 microeinsteins m^-2^s^-1^). At time zero, cyanophage strain S-PM2 was added to flask 1 at a multiplicity of infection of approximately 0.1 (= 1 mL of fresh lysate); 24 hours later, the same volume of cyanophage lysate was added to flask 2; flask 3 remained uninfected as a control. Flask 1 lysed (indicated by clearing of the culture) 3 days after initial infection, therefore, we predicted that virus mRNA would be detectable in flask 2 at this time point (24-hours prior to culture lysis). RNA and DNA were extracted from the cultures sampled at this time point as described below.

### Sampling to follow g20 gene expression during the cyanophage infection cycle

A 1 L culture of exponentially growing *Synechococcus *sp. WH7803 host cells was infected with cyanophage strain S-PM2 at a MOI of approximately 1 and incubated for 12 hours at 25°C under constant illumination. Duplicate 4 mL and 2 mL aliquots of infected cells (for RNA and DNA extraction respectively) were pelleted, snap frozen in liquid N_2 _then stored at -80°C at various time intervals over the 12 hour period. Frozen cell pellets were defrosted at 37°C and DNA and RNA were extracted as described below.

### Extraction of viral nucleic acid from infected host cells

RNA was extracted from 4 mL of pelleted cells using a Qiagen RNeasy^® ^Mini kit according to the manufacturer's instructions (Qiagen, West Sussex, UK). The protocol included a DNase treatment step. RNA was eluted in a final volume of 50 μL RNase-free sterile water. DNA was extracted from 2 mL of pelleted cells using a Qiagen DNeasy™ Tissue kit according to the manufacturer's instructions (Qiagen, West Sussex, UK). DNA was eluted in a final volume of 100 μL RNase-free sterile water. SMART assays [1,4] were conducted on 5 μL target nucleic acid, as described below.

### The SMART assay: isothermal amplification from specific target

Use of the SMART assay for the specific detection of cyanophage DNA has been described previously [4]. Target DNA was added to a mixture containing 2 μL 10× transcription buffer (Ambion, Austin, TX, USA), extension probe (5 fmol), template probe (1 fmol), facilitator probes 1 and 2 (100 fmol each) and ultra-pure, sterile, RNase-free water to a final volume of 15 μL. Samples were mixed, heated at 90°C for 3 min on a PTC-200™ thermal cycler (MJ Research, Waltham, MA, USA), ramped down to 41°C (0.1°C/s) and held at this temperature for 1 h. A 5 μL volume of solution containing dNTPs (5 μM each), NTPs (2 mM each) (both from Amersham Biosciences, Aylesbury UK), 4 U *Bst *(3' to 5'exo^-^) DNA polymerase (New England Biolabs, Beverly, MA, USA) and 240 U T7 RNA polymerase (Ambion) was then added, and the reaction was incubated at 41°C for an additional 2 h.

To amplify the RNA signal further, the samples were brought to room temperature before the addition of 20 fmol RNA amplification probe, followed by a mixture containing 4.5 μL 10× transcription buffer, dNTPs (50 μM each dNTP), NTPs (2 mM each NTP), 4 U *Bst *(3' to 5'exo^-^) DNA polymerase, 160 U T7 RNA polymerase, and ultra-pure, sterile, RNAse-free water to give a final volume of 17 μL. The samples were mixed and then incubated at 37°C for 2 h. The samples could be stored at -20°C before the signals were quantified.

### The SMART assay: capture and detection of the assay signal

The RNA signal was assayed by an Enzyme Linked OligoSorbent Assay (ELOSA). The RNA sequence includes regions for capture, via a biotinylated probe, and detection using a further probe linked to alkaline phosphatase (Fig. 1b). Biotinylated capture probe (0.9 pmol) and alkaline phosphatase-labelled probe (6 pmol) were added to each well of a streptavidin-coated Combiplate (Thermo Life Sciences, Hampshire, UK), in hybridisation buffer [50 mM Tris-HCl, pH 8.0, 1 M NaCl, 20 mM EDTA and 1% (w/v) BSA]. An aliquot (5–20 μL) of the sample to be quantified was then added, bringing the total volume to 150 μL per well. Samples were incubated at room temperature on a platform shaker at 300 rpm for 1 h. Unbound material was removed from wells by washing 4 times with 200 μL wash buffer [1× TBS/0.1% Tween-20], then once with 200 μL alkaline phosphatase substrate buffer (SCIL Diagnostics, Martinsried, Germany). Substrate (4-Nitrophenyl phosphate, Boehringer-Mannheim UK, Sussex, UK), at 5 mg/mL in substrate buffer, was then added (180 μL/well) and alkaline phosphatase activity was measured using a plate reader (Labsystems integrated EIA Management system, Thermo Life Sciences) pre-warmed at 37°C, reading absorbance at 405 nm every 2 minutes for 30 minutes. Rates of alkaline phosphatase activity for each sample were compared to a standard curve, generated using dilutions of a synthetic DNA oligonucleotide with the same sequence as the RNA product. This allowed the amount of RNA produced in each extension/transcription reaction to be calculated.

## Competing interests

SW is a former employee (1997–2001), and shareholder, of Cytocell Ltd. Patents for the SMART technology were held by Cytocell Ltd. However, since Cytocell Ltd has ceased to trade, there are no competing interests.

## Authors' contributions

SW participated in the design and co-ordination of the study, designed the specific probes, participated in interpretation of data and drafted the manuscript. MH generated and processed the samples, performed the SMART assays, and participated in interpretation of data. WW conceived the study, participated in its design and co-ordination, in the interpretation of data, and helped to draft the manuscript. All authors read and approved the final manuscript.

## References

[B1] Wharam SD, Marsh P, Lloyd JS, Ray TD, Mock GA, Assenberg R, McPhee JE, Brown P, Weston A, Cardy DLN (2001). Specific detection of DNA and RNA targets using a novel isothermal nucleic acid amplification assay based on the formation of a three-way junction structure. Nucleic Acids Research.

[B2] Levi K, Bailey C, Bennett A, Marsh P, Cardy DL, Towner KJ (2003). Evaluation of an Isothermal Signal Amplification Method for Rapid Detection of Methicillin-Resistant Staphylococcus aureus from Patient-Screening Swabs. Journal of Clinical Microbiology.

[B3] Brown DFJ, Edwards DI, Hawkey PM, Morrison D, Ridgway GL, Towner KJ, Wren MWD, on behalf of the Joint Working Party of the British Society for Antimicrobial Chemotherapy HISaICNA (2005). Guidelines for the laboratory diagnosis and susceptibility testing of methicillin-resistant Staphylococcus aureus (MRSA). Journal of Antimicrobial Chemotherapy.

[B4] Hall MJ, Wharam SD, Weston A, Cardy DLN, Wilson WH (2002). Use of signal-mediated amplification of RNA technology (SMART) to detect marine cyanophage DNA. Biotechniques.

[B5] Suttle CA, Whitton BA, Potts M (2000). Cyanophages and their role in the ecology of cyanobacteria. The ecology of cyanobacteria: Their diversity in time and space.

[B6] Suttle CA, Chan AM (1993). Marine Cyanophages Infecting Oceanic and Coastal Strains of *Synechococcus *– Abundance, Morphology, Cross-Infectivity and Growth-Characteristics. Marine Ecology-Progress Series.

[B7] Suttle CA, Chan AM (1994). Dynamics and Distribution of Cyanophages and Their Effect On Marine *Synechococcus *spp. Applied and Environmental Microbiology.

[B8] Waterbury JB, Valois FW (1993). Resistance to Cooccurring Phages Enables Marine *Synechococcus *Communities to Coexist With Cyanophages Abundant in Seawater. Applied and Environmental Microbiology.

[B9] Wilson WH, Joint IR, Carr NG, Mann NH (1993). Isolation and molecular characterization of five marine cyanophages propagated on *Synechococcus *sp strain WH7803. Applied and Environmental Microbiology.

[B10] Waterbury JB, Watson SW, Valois FW, Franks DG, Platt T, Li WKW (1986). Biological and ecological characterisation of the marine unicellular cyanobacterium *Synechococcus*. Photosynthetic picoplankton.

[B11] Hennes KP, Suttle CA, Chan AM (1995). Fluorescently Labeled Virus Probes Show That Natural Virus Populations Can Control the Structure of Marine Microbial Communities. Applied and Environmental Microbiology.

[B12] Fuhrman JA (1999). Marine viruses and their biogeochemical and ecological effects. Nature.

[B13] Hewson I, Govil SR, Capone DG, Carpenter EJ, Fuhrman JA (2004). Evidence of *Trichodesmium *viral lysis and potential significance for biogeochemical cycling in the oligotrophic ocean. Aquatic Microbial Ecology.

[B14] Wilhelm SW, Suttle CA (1999). Viruses and Nutrient Cycles in the Sea – Viruses play critical roles in the structure and function of aquatic food webs. Bioscience.

[B15] Wilson WH, Fuller NJ, Joint IR, Mann NH, Charpy L, Larkum AWD (1999). Analysis of cyanophage diversity and population structure in a south-north transect of the Atlantic Ocean. Marine Cyanobacteria.

[B16] Wilson WH, Fuller NJ, Joint IR, Mann NH, Bell CR, Brylinsky M, Johnson-Green P (2000). Analysis of cyanophage diversity in the marine environment using denaturing gradient gel electrophoresis. Microbial Biosystems: New Frontiers Proceedings of the 8th International Symposium on Microbial Ecology Atlantic Canada Society for Microbial Ecology.

[B17] McDaniel L, Houchin LA, Williamson SJ, Paul JH (2002). Plankton blooms – Lysogeny in marine *Synechococcus*. Nature.

[B18] Ortmann AC, Lawrence JE, Suttle CA (2002). Lysogeny and lytic viral production during a bloom of the cyanobacterium *Synechococcus *spp. Microbial Ecology.

[B19] Zhong Y, Chen F, Wilhelm SW, Poorvin L, Hodson RE (2002). Phylogenetic diversity of marine cyanophage isolates and natural virus communities as revealed by sequences of viral capsid assembly protein gene g20. Applied and Environmental Microbiology.

[B20] Muhling M, Fuller NJ, Millard A, Somerfield PJ, Marie D, Wilson WH, Scanlan DJ, Post AF, Joint I, Mann NH (2005). Genetic diversity of marine Synechococcus and co-occurring cyanophage communities: evidence for viral control of phytoplankton. Environmental Microbiology.

[B21] Fuller NJ, Wilson WH, Joint IR, Mann NH (1998). Occurrence of a sequence in marine cyanophages similar to that of T4 g20 and its application to PCR-based detection and quantification techniques. Applied and Environmental Microbiology.

[B22] Hambly E, Tetart F, Desplats C, Wilson WH, Krisch HM, Mann NH (2001). A conserved genetic module that encodes the major virion components in both the coliphage T4 and the marine cyanophage S-PM2. Proceedings of the National Academy of Sciences of the United States of America.

[B23] Chen F, Lu JR (2002). Genomic sequence and evolution of marine cyanophage P60: a new insight on lytic and lysogenic phages. Applied and Environmental Microbiology.

[B24] Mann NH, Clokie MRJ, Millard A, Cook A, Wilson WH, Wheatley PJ, Letarov A, Krisch HM (2005). The genome of S-PM2, a "photosynthetic" T4-type bacteriophage that infects marine Synechococcus strains. Journal of Bacteriology.

[B25] Sullivan MB, Coleman ML, Weigele P, Rohwer F, Chisholm SW (2005). Three Prochlorococcus cyanophage genomes: Signature features and ecological interpretations. Plos Biology.

[B26] Lindell D, Sullivan MB, Johnson ZI, Tolonen AC, Rohwer F, Chisholm SW (2004). Transfer of photosynthesis genes to and from Prochlorococcus viruses. Proceedings of the National Academy of Sciences of the United States of America.

[B27] Mann NH, Cook A, Millard A, Bailey S, Clokie M (2003). Marine ecosystems: Bacterial photosynthesis genes in a virus. Nature.

[B28] Sullivan MB, Lindell D, Lee JA, Thompson LR, Bielawski JP, Chisholm SW (2006). Prevalence and evolution of core photosystem II genes in marine cyanobacterial viruses and their hosts. Plos Biology.

[B29] Lindell D, Jaffe JD, Johnson ZI, Church GM, Chisholm SW (2005). Photosynthesis genes in marine viruses yield proteins during host infection. Nature.

[B30] Clokie MRJ, Shan JY, Bailey S, Jia Y, Krisch HM, West S, Mann NH (2006). Transcription of a 'photosynthetic' T4-type phage during infection of a marine cyanobacterium. Environmental Microbiology.

[B31] Clokie MRJ, Mann NH (2006). Marine cyanophages and light. Environmental Microbiology.

[B32] Black LW, Showe MK, Steven AC, Karam JD, Drake JW, Kreuzer KN, Mosig G, Hall DH, Eiserling FA, Black LW, Spicer EK, Kutter E, Carlson K, Miller ES (1994). Morphogenesis of the T4 Head. Molecular Biology of Bacteriophage T4.

[B33] Dorigo U, Jacquet S, Humbert JF (2004). Cyanophage Diversity, Inferred from g20 Gene Analyses, in the Largest Natural Lake in France, Lake Bourget. Applied and Environmental Microbiology.

[B34] Marston MF, Sallee JL (2003). Genetic Diversity and Temporal Variation in the Cyanophage Community Infecting Marine Synechococcus Species in Rhode Island's Coastal Waters. Applied and Environmental Microbiology.

[B35] Sandaa RA, Larsen A (2006). Seasonal Variations in Virus-Host Populations in Norwegian Coastal Waters: Focusing on the Cyanophage Community Infecting Marine Synechococcus spp. Applied and Environmental Microbiology.

[B36] Short CM, Suttle CA (2005). Nearly Identical Bacteriophage Structural Gene Sequences Are Widely Distributed in both Marine and Freshwater Environments. Applied and Environmental Microbiology.

[B37] Wang K, Chen F (2004). Genetic diversity and population dynamics of cyanophage communities in the Chesapeake Bay. Aquatic Microbial Ecology.

[B38] Wilhelm SW, Carberry MJ, Eldridge ML, Poorvin L, Saxton MA, Doblin MA (2006). Marine and Freshwater Cyanophages in a Laurentian Great Lake: Evidence from Infectivity Assays and Molecular Analyses of g20 Genes. Applied and Environmental Microbiology.

[B39] Wilson WH, Carr NG, Mann NH (1996). The effect of phosphate status on the kinetics of cyanophage infection in the oceanic cyanobacterium *Synechococcus *sp WH7803. Journal of Phycology.

[B40] Mathews CK, Karam JD, Drake JW, Kreuzer KN, Mosig G, Hall DH, Eiserling FA, Black LW, Spicer EK, Kutter E, Carlson K, Miller ES (1994). An overview of the T4 developmental program. Molecular Biology of Bacteriophage T4.

[B41] Black LW, Calendar R (1988). DNA packaging in dsDNA Bacteriophages. The Bacteriophages.

